# Proliferative, pro-inflammatory, and angiogenesis regulator gene expression profile defines prognosis in different histopathological subtypes of nodal peripheral T-cell lymphoma

**DOI:** 10.18632/oncotarget.27098

**Published:** 2019-08-27

**Authors:** Luís Alberto de Pádua Covas Lage, Débora Levy, Flávia Dias Xavier, Diego Cândido Reis, Renata de Oliveira Costa, Marianne Castro Gonçalves, Vanderson Rocha, Maria Cláudia Nogueira Zerbini, Juliana Pereira

**Affiliations:** ^1^ Department of Hematology, Hemotherapy and Cell Therapy, Faculdade de Medicina da Universidade de São Paulo, São Paulo, SP, Brazil; ^2^ Laboratory of Medical Investigation in Genetics and Molecular Hematology (LIM-31), Universidade de São Paulo, SãoPaulo, Brazil; ^3^ Department of Hematology and Hemotherapy, Faculdade de Medicina da Universidade de Brasília, Brasília, Brazil; ^4^ Medical Sciences Discipline, Faculdade de Medicina da Universidade de São Paulo, São Paulo, Brazil; ^5^ Department of Hematology and Hemotherapy Discipline, Faculdade de Ciências Médicas de Santos/Centro Universitário Lusíada, Santos, Brazil; ^6^ Departamento of Pathology, Faculdade de Medicina da Universidade de São Paulo, São Paulo, Brazil; ^7^ President Fundação Pró-Sangue, Churchill Hospital, Oxford University, Oxford, UK

**Keywords:** peripheral T-cell lymphoma, gene expression analysis, cell cycle regulation, inflammation, prognosis

## Abstract

Nodal peripheral T-cell lymphoma (PTCL) is an aggressive and heterogeneous malignancy with poor prognosis. We studied the prognostic impact of the expression profile of genes related to cell proliferation (*CCNA2*, *TOP2A*, and *CHEK1*), pro-inflammatory activity (*NFkB1* and *IKBkB*), and angiogenesis (*VEGF1*) in nodal PTCL outcomes, as well as the ability of this genomic panel to discriminate different histological subtypes. We investigated the relative expression of regulator genes in 63 nodal PTCL patients. CCNA2, TOP2A, CHEK1, and NF-kB1 proteins were also assessed by immunohistochemistry. The median patient age was 47 years, 57.1% were male, 34.9% were diagnosed with PTCL-NOS, 28.6% with ALK-/ALCL, 22.2% with ALK+/ALCL, and 14.3% with AITL. The proliferative genes were associated with worse 3-year OS and PFS in PTCL-NOS and better 3-year PFS in ALK-/ALCL. Expression of CCNA2≥median and overexpression of CHEK1 protein (HR 3.793; *p* = 0.007) were associated with worse OS for all the cohort of nodal PTCL (HR 1.418; *p* = 0.001). The genomic expression profile tested in this study was not able to discriminate the different subtypes of nodal PTCL, although it showed a distinct prognostic significance between PTCL-NOS and ALCL-ALK. Overexpression of the CCNA2 gene and CHEK1 protein were associated with poor prognosis in the total nodal PTCL cohort.

## INTRODUCTION

Peripheral T-cell lymphoma (PTCL) belongs to a heterogeneous group of rare neoplasms of mature T lymphocytes, representing 10% to 15% of lymphoid malignancies [1]. The incidence of these lymphomas varies according to the geographic region; with a higher frequency in Southeast Asia and in Central and South America, where they represent up to 20% to 25% of all non-Hodgkin lymphomas (NHLs) [2]. The most recent World Health Organization (WHO) classification of lymphoid and hematopoietic tissue neoplasms divides PTCL according to clinical aspects into predominantly nodal, extra nodal, primary cutaneous, and disseminated or leukemic subtypes [3].

The predominantly nodal PTCL represents 60% to 70% of all PTCL, and includes peripheral T-cell lymphoma, not otherwise specified (PTCL-NOS), angioimmunoblastic T-cell lymphoma (AITL), and anaplastic large cell lymphoma (ALCL), which is further subdivided intoanaplasticlymphoma kinase (ALK) positive and negative variants [4]. However, overlapping morphological and immunophenotypical aspects among the different subtypes of nodal PTCL are not uncommon, leading to inappropriate diagnosis [3].

In some cases, cytogenetic and molecular features may also help to individualize a subtype of PTCL, but this kind of analysis is not routinely available in most centers. Therefore, novel biological markers are needed to better differentiate PTCL subtypes. Moreover, few biological prognostic markers have been described for PTCL, such as ALK expression associated witht(2;5)(p23; q35) involving the gene *ALK* on chromosome 2, and ALCL [5]. Similarly, *IRF4* and *DUSP22* rearrangement are associated with ALK-negative ALCL and deletion of chromosome 6q in nasal type natural killer lymphoma [6, 7].

Except for ALK-positive ALCL, nodal PTCL patients have short survival, with a 5-year overall survival (OS) of 30% to 40%. This is partially related to the presence of worse clinical and laboratory features at diagnosis in these patients. Nodal PTCL patients are usually older, with high levels of dehydrogenase lactic enzyme (LDH), high risk of International Prognostic Index (IPI), and bone marrow infiltration. Nodal PTCLs may exhibit high P-glycoprotein (gpP) expression, which is associated with multiple drug resistance (MDR) phenotype and *p53* mutation [8]. The main prognostic indexes used for nodal PTCL are the IPI [9], and the prognostic index for PTCL(PIT) [10].

To better characterize the molecular heterogeneity and physiopathology of nodal PTCLs, and to provide more rationality in its treatment, we evaluated the prognostic impact of cyclin A2 (*CCNA2*), DNA topoisomerase Iiα(*TOP2A*), checkpoint kinase 1 (*CHEK1*), factor nuclear kappa B (*NF-kB1*), inhibitor of nuclear factor kappa B kinase subunit β (*IKBkB*), and vascular endothelial growth factor 1 (*VEGF1*)gene expression and CCNA2, TOP2A, CHEK1 and NF-kB1 proteins in nodal PTCL at a single cancer treatment center in Brazil. This is a hypothesis-driven study, and the genes chosen were selected based on the findings of Cuadros *et al*. [11] which implicated such genes in the biology and pathogenesis of these neoplasms. We also evaluated the potential of these biomarkers to discriminate different histological subtypes of nodal PTCL.

## RESULTS

Of the 94 patients initially enrolled in this study, 7 (7.5%) were excluded due to unavailability of medical records or after histology revision. Two cases previously diagnosed as nodal PTCL were changed to primary cutaneous ALCL and another to adult T-cell leukemia/lymphoma. Therefore, clinical and epidemiological data were available for 87 (92.5%) patients, but 24 (27.5%) were excluded due to lack of paraffin blocks and failure at RNA extraction or cDNA amplification, remaining 63 (72.5%) patients available for gene expression study and 54 (62.6%) for IHC analysis.

### Patients

The median age of the 63 patients with nodal PTCL was 47 years (range 18–81 years). Fifty-seven percent (*n* = 36) of the patients in this study were male. The PTCL-NOS subtype was predominant (*n* = 22, 34.9%), followed by ALK-negative ALCL (*n* = 18, 28.6%), ALK-positive ALCL (*n* = 14, 22.2%), and AITL (*n* = 9, 14.3%; Table 1). The clinical characteristics of the patients are provided in Table 2. ALK-positive ALCL and ALK-negative ALCL cases were comprised predominantly of young patients; 65% (9/14) of ALK-positive ALCL and 50% (9/18) ALK-negative ALCL patients were < 40 years of age. ALK-positive ALCL presented unfavorable features, such as ECOG ≥ 2 in 71.4% of cases and high-risk adapted IPI/PIT in 50% of cases. However, ALK-negative ALCL presented ECOG ≥ 2 in 44.4% (8/18) of cases, and 50% (9/18) were classified as an adapted IPI of high risk.

**Table 1:** Characteristics of all PTCL patients

Characteristic	Total (*N* = 87)	Included in gene analysis (*N* = 63)
Gender		
Female	35 (40.2)	27 (42.9)
Male	52 (59.8)	36 (57.1)
Median age, years	49 (14-84)	47 (16–81)
>60 years	26 (29.9)	14 (22.2)
Histopathological variant		
ALCL/ALK+	18 (20.7)	14 (22.2)
ALCL/ALK-	29 (33.3)	18 (28.6)
PTCL-NOS	30 (34.5)	22 (34.9)
AITL	10 (11.5)	09 (14.3)
Elevated LDH		
< UVN	34 (39.1)	21 (33.3)
≥ UVN	53 (60.9)	42 (66.7)
ECOG		
<2	34 (39.1)	23 (39.1)
≥2	53 (60.9)	40 (60.9)
Bone marrow		
Positive	10 (11.5)	08 (12.7)
Negative	67 (70.0)	48 (76.9)
Unavailable	10 (11.5)	07 (11.1)
Extranodal sites ≥ 2	38 (43.7)	27 (42.9)
Bulky		
Yes	35 (40.2)	24 (30.1)
No	52 (59.8)	39 (69.9)
Clinical stage		
Early (I/ II)	22 (25.3)	12 (19.0)
Advanced (III/IV)	65 (74.7)	51 (81.0)
Adapted IPI		
Low (LR + IL)	34 (39.1)	24 (38.1)
High (HI+ HR)	53 (60.9)	39 (61.9)
Adapted IPI-T	(N=85)2	(*N* = 56)2
Low (LR + IL)	31 (36.5)	29 (51.8)
High (HI + HR)	54 (53.5)	34 (60.7)
Radiotherapy		
No	61 (70.1)	46 (73)
Yes	26 (29.9)	17 (27.0)
Disease response		
CR	36 (41.4)	25 (39.7)
PR	05 (05.7)	04 (06.3)
Progression and/or death	46 (52.9)	34 (54.0)
ASCT		
No	58 (66.7)	41 (65.1)
Yes	29 (33.3)	22 (34.9)
Treatment		
CHOP	45 (51.7)	32 (50.8)
CHOEP	19 (21.8)	15 (23.8)
Other	13 (14.9)	11 (17.5)
None	10 (11.0)	05 (07.9)
Death cause	46 (52.9)	34 (54.0)
Progression disease4	26 (56.5)	22 (64.7)
Infection4	24 (52.2)	15 (44.1)
Second neoplasm4	04 (08.7)	04 (11.7)

Data are presented as *n* (%) or median (range). ^1^10 and 3 patients excluded due to missing data. ^2^10 patients excluded due to missing data, 8 cases with high-risk adjusted IPI-T with missing data. *N* = 85 considered for the subgroup analysis. ^3^Two acute myeloid leukemia and 2 solid tumors (pancreatic adenocarcinoma and lung squamous cell carcinoma). ^4^Percentage calculated among deaths. ALCL: anaplastic large cell lymphoma; ALK: anaplastic lymphoma kinase; PTCL-NOS: peripheral T cell lymphoma, not otherwise specified; AITL: angioimmunoblastic T-cell lymphoma; LDH: lactic dehydrogenase; ECOG: Eastern Cooperative Oncology Group; IPI: International Prognostic Index; IPI-T: International Prognostic Index for T lymphoma; LR: low risk; IL: intermediate-low; HI: high-intermediate; HR: high risk; CR: complete response; PR: partial response; ASCT: autologous hematopoietic stem cell transplantation; CHOP: cyclophosphamide, doxorubicin, vincristine and prednisone; CHOEP: cyclophosphamide, doxorubicin, vincristine, etoposide, and prednisone.

**Table 2:** Clinical and laboratory features by histopathological subgroups

Characteristic	AITL (*n* = 9)	ALK-ALCL (*n* = 18)	ALK+ ALCL (*n* = 14)	PTCL-NOS (*n* = 22)
Gender				
Female	2 (22.2)	7 (38.8)	7 (50.0)	11 (50.0)
Male	7 (77.8)	11 (61.2)	7 (50.0)	11 (50.0)
Age				
≤ 60 years	5 (55.6)	14 (77.7)	12 (85.7)	16 (72.7)
> 60 years	4 (44.4)	4 (22.3)	2 (14.3)	6 (27.3)
LDH				
< UVN	1 (11.1)	8 (44.4)	6 (42.8)	6 (27.3)
≥ UVN	8 (88.9)	10 (55.6)	8 (57.2)	16 (72.7)
ECOG				
<2	2 (22.2)	10 (55.6)	4 (28.6)	7 (31.8)
≥2	7 (77.8)	8 (44.4)	10 (71.4)	15 (68.2)
Bone marrow				
Positive	1 (11.1)	4 (22.3)	2 (14.3)	2 (9.1)
Negative	8 (88.9)	14 (77.7)	12 (85.7)	20 (90.9)
Bulky				
Yes	0 (0.0)	10 (55.6)	7 (50.0)	7 (31.8)
No	9 (100.0)	8 (44.4)	7 (50.0)	15 (68.2)
Clinical stage				
Early (I/II)	0 (0.0)	1 (5.6)	0 (0.0)	2 (9.1)
Advanced (III/IV)	9 (100.0)	17 (94.4)	14 (100.0)	20 (90.9)
Adapted IPI				
Low (LR+IL)	2 (22.2)	9 (50.0)	7 (50.0)	6 (27.3)
High (HI+HR)	7 (77.8)	9 (50.0)	7 (50.0)	16 (72.7)
Adapted IPI-T				
Low (LR+IL)	2 (22.2)	9 (50.0)	7 (50.0)	5 (22.7)
High (HI+HR)	7 (77.8)	9 (50.0)	7 (50.0)	17 (77.3)
Radiotherapy				
No	0 (0.0)	13 (72.2)	9 (64.3)	16 (72.7)
Yes	9 (100.0)	6 (27.8)	5 (35.7)	6 (27.3)
ASCT				
No	8 (88.9)	7 (38.8)	10 (71.4)	17 (68.2)
Yes	1 (11.1)	12 (61.2)	4 (28.6)	5 (31.8)
Death				
Yes	6 (66.6)	4 (22.3)	7 (50.0)	17 (68.2)
No	3 (33.4)	14 (77.7)	7 (50.0)	5 (31.8)
Death cause1	(*n* = 6)	(*n* = 4)	(*n* = 7)	(*n* = 17)
Progression disease	4 (66.6)	2 (50.0)	5 (71.4)	11 (64.8)
Infection	2 (33.4)	2 (50.0)	2 (28.6)	3 (17.6)
Other	0 (0.0)	0 (0.0)	0 (0.0)	3 (17.6)
Treatment				
CHOP	5 (55.6)	10 (55.6)	8 (57.2)	9 (40.9)
CHOEP	1 (11.1)	4 (22.2)	3 (21.4)	6 (27.2)
Other	3 (33.3)	4 (22.2)	3 (21.4)	7 (31.9)
First line response				
CR	4 (44.4)	11 (61.1)	7 (50.0)	7 (31.9)
PR	0 (0.00)	3 (16.7)	3 (21.4)	2 (9.1)
PD plus death	5 (55.6)	4 (22.2)	4 (28.6)	13 (59.0)

Data are presented as *n* (%). ^1^Percentage calculated among deaths. AITL: angioimmunoblastic T cell lymphoma; ALCL: anaplastic large cell lymphoma; ALK: anaplastic lymphoma kinase; PTCL-NOS: peripheral T cell Lymphoma, not otherwise specified; LDH: lactic dehydrogenase; UVN: upper value of normality; ECOG: Eastern Cooperative Oncology Group; CS: clinical stage; IPI: International Prognostic Index; IPI-T: International Prognostic Index for T-cell lymphoma; LR: low risk; IL: intermediate-low; HI: high-intermediate; HR: high risk; ASTC: autologous hematopoietic stem cell transplantation; CHOP: cyclophosphamide, doxorubicin, vincristine, and prednisone; CHOEP: cyclophosphamide, doxorubicin, vincristine, etoposide, and prednisone; CR: complete response; PR: partial response.

The overall response rate (ORR) was 60.3% (38/63), with 46% (29/63) CR and 14.3% (9/63) PR, whereas 7.9% (5/63) were primary refractory to the treatment. Thirty-four (54%) patients died, 20 (31.7%) during the first line chemotherapy period. The most common cause of death was related to lymphoma and occurred under progression disease (PD) status. Twenty (34.9%) patients underwent ASCT in first or second remission.

**Figure 1:**
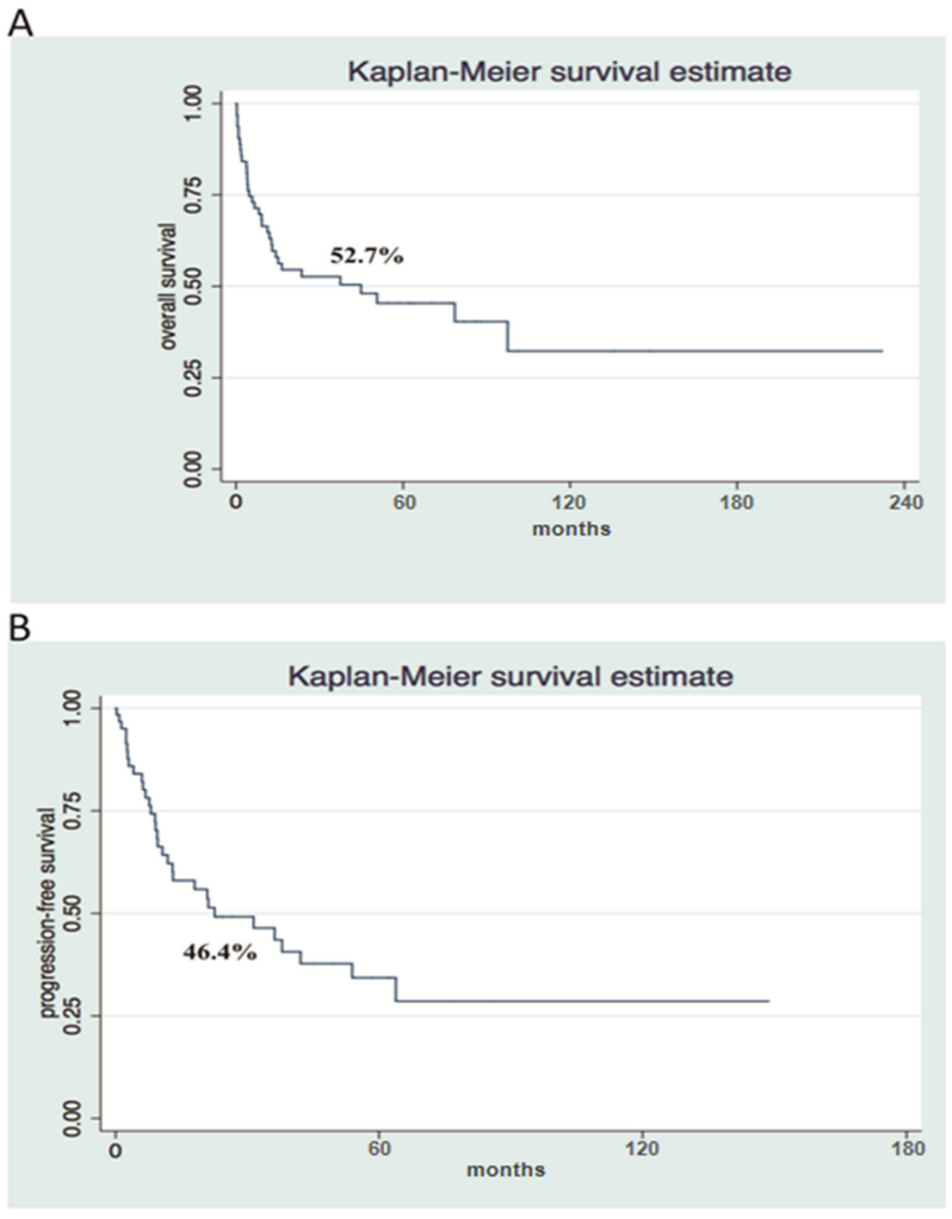
Survival analysis shows poor Overall Survival and Progression Free Survival in a cohort of patients with nodal PTCL. Kaplan-Meier Overall Survival (**A**) and Progression-free Survival (**B**) curves at 3-years (*n* = 63).

**Figure 2:**
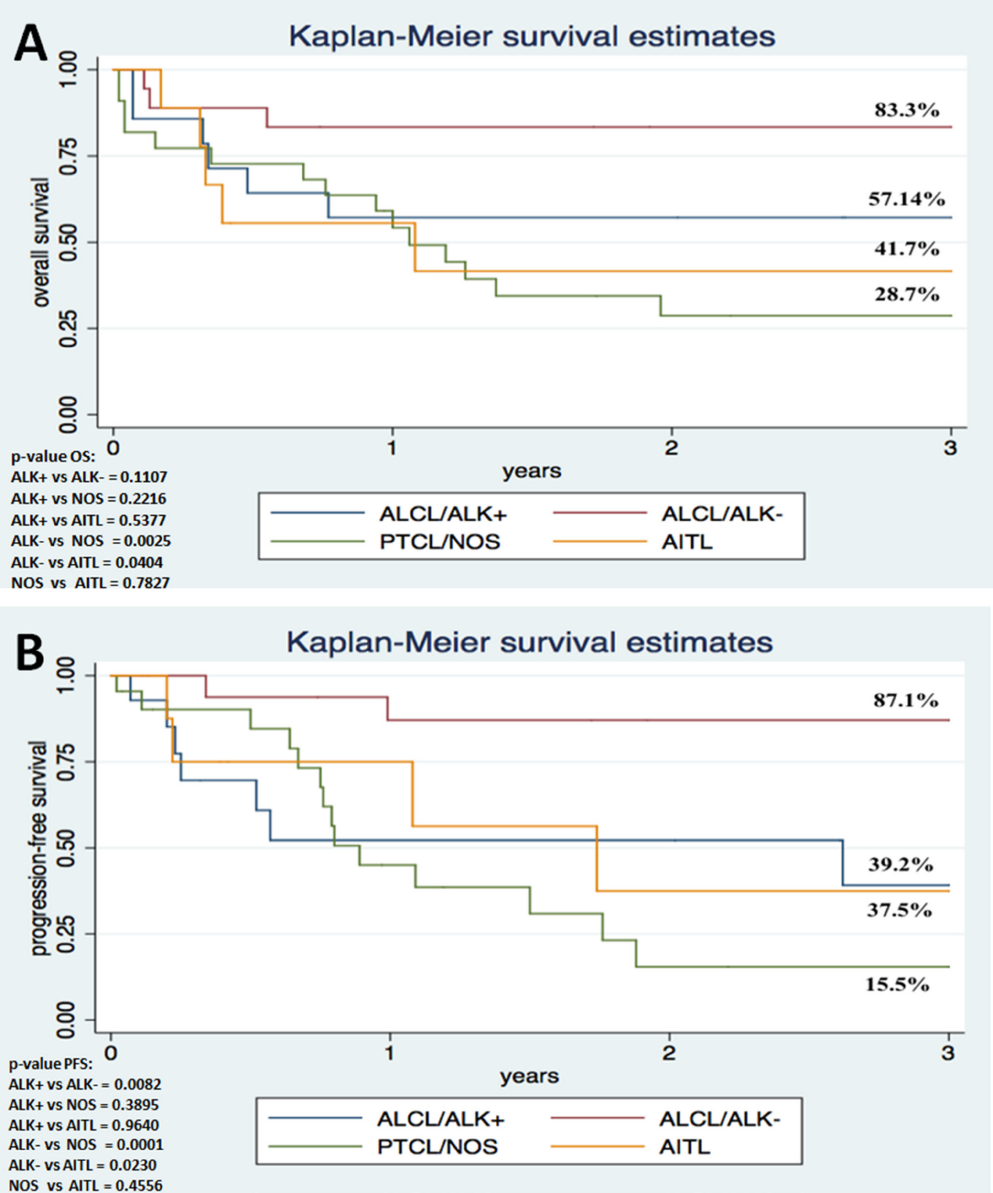
Survival analysis shows poor outcome in ALCL patients with ALK1 immunoexpression in comparison to individuals lacking ALK1 expression. Kaplan-Meier Overall Survival (**A**) and Progression-free Survival (**B**) curves according to histopathological variant (*N* = 63).

The median OS was 44.88 months (0.29-232 months), and 3-year OS was 52.7% (Figure 1). The median PFS was 22.52 months (0.20-148 months) and 3-year PFS was 46.4% (Figure 1). The 3-year OS and PFS were 83.30% and 87.05% for ALK-negative ALCL, 57.14% and 39.17% for ALK-positive ALCL, 41.67% and 37.50% for AITL, and 28.72% and 15.45% for PTCL-NOS, respectively (Figure 2).

### Gene expression analysis

**Figure 3:**
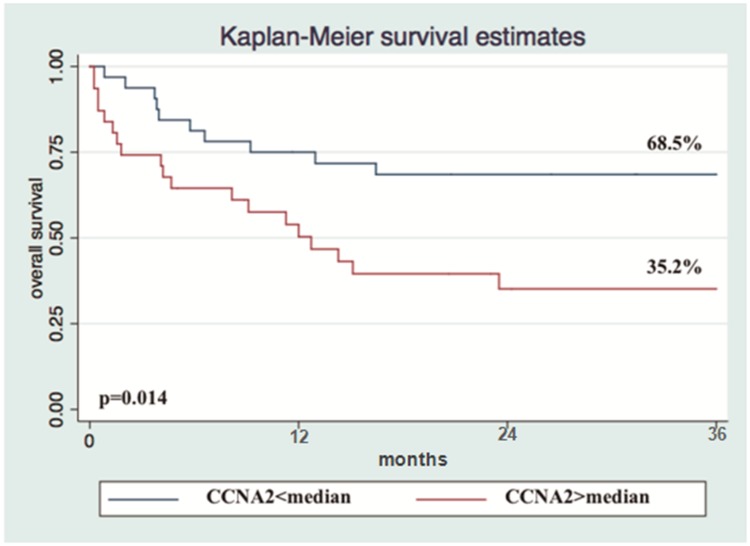
Overexpression of CCNA2 gene is a determinant marker of poor prognosis in nodal PTCL. Kaplan-Meier Overall Survival curve according to CCNA2 gene expression (*n* = 63).

The median relative expression in the 63 nodal PTCL patients was 2.00 for *CCNA2*, 4.22 for *TOP2A*, 6.20 for *CHEK1*, 0.49 for *VEGF1*, 10.59 for *NFkB1,* and 8.35 for *IKBkB*. Gene expression was analyzed as a qualitative categorical variable, taking into account values < or ≥ the median. In univariate analysis, *CCNA2* expression ≥ median was associated with worse 3-year OS for the whole cohort (OS 68.5% for *CCNA2*< median and 35.15% for *CCNA2* ≥ median; hazard ratio [HR] 2.618, 95% CI 1.2137-5.6497, *p* = 0.014) (Figure 3).

None of the six genes exhibited a prognostic correlation with OS and PFS for ALK-positive ALCL or AITL. Overexpression of *VEGF1* was associated with worse 3-year PFS for ALK-negative ALCL (100% for *VEGF1* < median and 76,19% for *VEGF1 ≥* median; HR 1.74,0.869–3.760, *p* = 0.04). For PTCL-NOS, *CCNA2* (85.7% vs. 0%; HR 25.193, 95% CI 3.140–202.120, *p* < 0.002), *TOP2A* (85.7% vs. 0%; HR 17.455, 95% CI 2.191-139.039, *p* = 0.007), *CHEK1* (75% vs. 0%; HR 9.490, 95% CI 2.035–44.252, *p* = 0.004), and *IKBkB* (49.1% vs. 9.1%; HR 3.763, 95% CI 1.255–11.281, *p* = 0.018) ≥ median were associated with worse 3-year OS. In the subgroup of ALK-negative ALCL, *CCNA2* and *CHEK1* ≥ median were associated with better 3-year PFS (71.4% for *CCNA2*< median and 100% for *CCNA2* ≥ median [*p* = 0.02] and 75% for *CHEK1*< median and 100% for *CHEK1* ≥ median [*p* = 0.001]).

**Figure 4:**
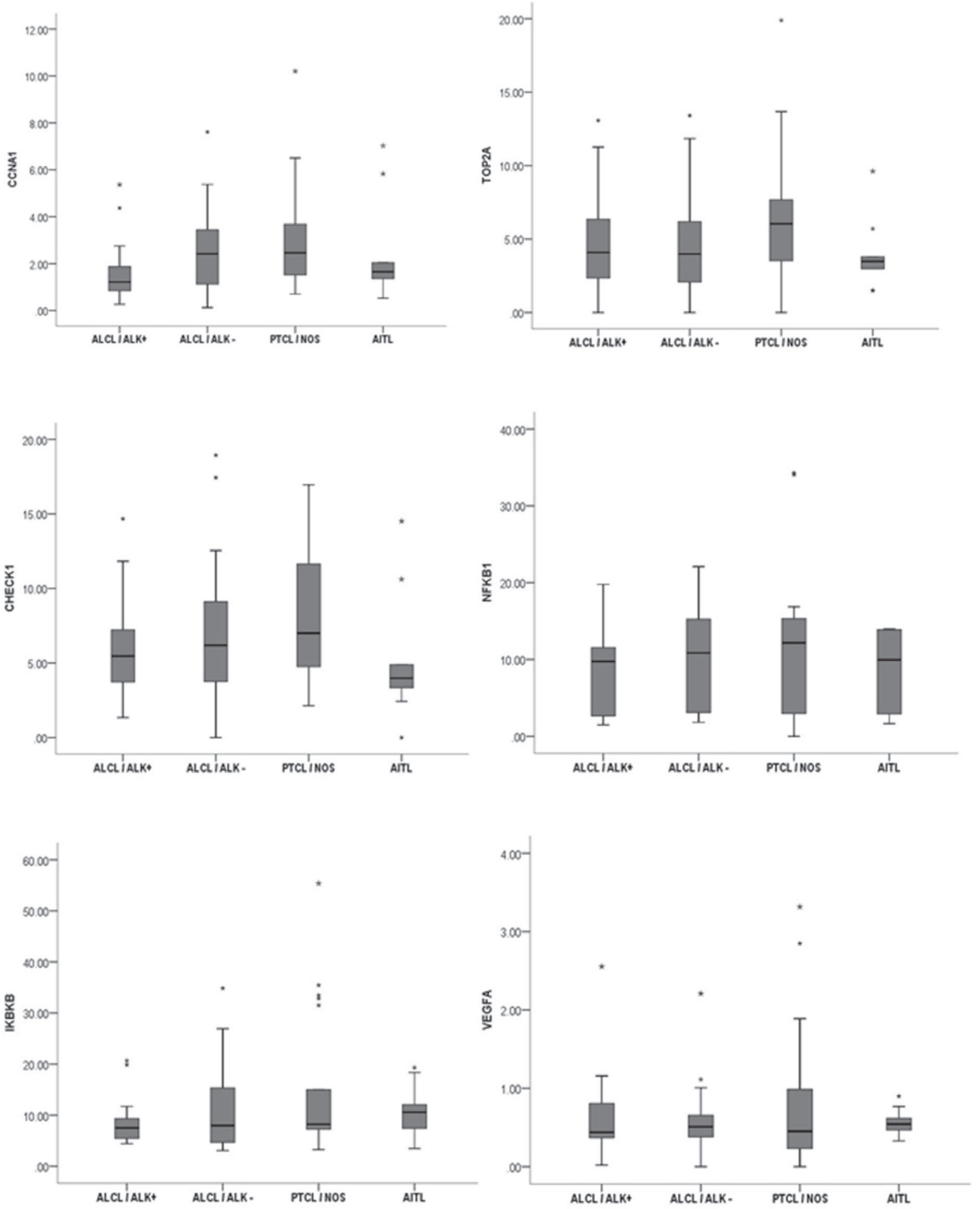
The analyzed gene panel was not able to distinguish different histopathological variants of nodal PTCL. Distribution pattern of the median intensity of gene expression according to morphological variant of nodal PTCL.

The analyzed gene panel was not able to discriminate any specific histological subtypes of nodal PTCL. However, all genes, except *VEGF1*, demonstrated a typical and linear pattern of distribution of median expression intensity in decreasing order: PTCL-NOS > ALK- ALCL > ALK+ ALCL (Figure 4).

### Immunohistochemistry

In the IHC performed in 54 patients, the cut-off of positivity was 50% for CCNA2, 70% for TOP2A, 30% for NF-kB1, and 60% for CHEK1. For all patients, univariate analysis showed that CHEK1 protein ≥ 60% was associated with worse 3-year OS (37,57% vs. 79,69%, HR 4.062, 95% CI 1.363–12.104; *p* = 0.012) and TOP2A ≥ 70% was associated with better 3-year PFS (76.6%vs 28.8%, HR 0.272, 95% CI 0.0785–0.9450; *p* = 0.04) than CHEK1 < 60% and TOP2A < 70%, respectively.

### Multivariate analysis

In multivariate analysis, *CCNA2* expression ≥ median (HR 1.418,95% CI 1.148–1.751; *p* = 0.001), CHEK1 protein ≥ 60% (HR3.793, 95% CI 1.446–9.945; *p* = 0.007), and ECOG ≥ 2 (HR 6.506, 95% CI 2.070–20.442; *p* = 0.001), IPI high-risk and intermediate-high (HR 2.042, 95% CI 1.148–3.632; *p* = 0.015) were associated with worse 3-year OS for the whole cohort. ECOG ≥ 2 (HR 4.594, 95% CI 1.614–13.075; *p* = 0.004) and IPI high-risk and intermediate-high (HR: 10.020, 95% CI 1.967–30.567; *p* = 0.006) were found to be an independent predictor of PFS at 3 years. The main results of multivariate analysis are summarized in Table 3.

**Table 3:** Main results of multivariate analysis

Variable	HR	95% CI	*P*-value	Outcome
CCNA2 ≥ median	1.418	1.148–1.751	0.001	Poor 3-year OS
CHEK1 ≥ 60%	3.793	1.446–9.945	0.007	Poor 3-year OS
ECOG ≥ 2	6.506	2.070–20.442	0.001	Poor 3-year OS
IPI^*^	2.042	1.148–3.632	0.015	Poor 3-year OS
PIT^*^	2.792	1.213–6.425	0.016	Poor 3-year OS
IPI^*^	10.020	1.967–30.567	0.006	Poor 3-year PFS
ECOG ≥ 2	4.594	1.614–13.075	0.004	Poor 3-year PFS

HR: Hazard ratio; CI: Confidence interval; CCNA2: CCNA2 gene expression; CHEK1: CHEK1 protein expression; ECOG: Eastern Cooperative Oncology Group; OS: Overall survival; PFS: Progression-free survival. IPI^*^: International Prognostic Index high-risk and intermediate-high. PIT^*^: Prognostic Index of Peripheral T-cell Lymphomas high-risk and intermediate-high.

## DISCUSSION

In this study, we showed that overexpression of *CCNA2* gene and CHEK1 protein is associated with worse OS for nodal PTCL independent of the IPI, and ECOG ≥ 2 is the main independent variable for 3-year PFS. Our results are partly in agreement with those obtained by Cuadros *et al.* [11], who analyzed the gene expression profiles of 35 nodal PTCL patients (23 PTCL-NOS and 12 AITL) using two different microarray platforms.

These authors identified five distinct clusters comprising genes related to cell proliferation, inflammatory processes, and B-cell and T-cell lineages. They observed that a predominance of genes related to cell proliferation and cell cycle control, such as *CCNA*, *CCNB*, *TOP2A,* and *PCNA,* were significantly associated with poor prognosis [11]. However, in our study, univariate analysis showed that TOP2A protein ≥ 70% was associated with better OS for the whole cohort of nodal PTCL patients and worse prognosis for the PTCL-NOS subgroup. This contradictory result may be explained by the differences between the studies; Cuadros *et al*. studied only PTCL-NOS and AITL histological subgroups, whereas we analyzed more cases and included patients with an ALCL diagnosis.

The *TOP2A* gene encodes the enzyme topoisomerase 2 alpha, which is a common target of anticancer therapy (e.g., etoposide). This enzyme participates in the topological arrangement of DNA during RNA transcription. Consequently, TOP2A inhibition may disrupt the DNA double helix during the S phase of the cell cycle and subsequently induce apoptosis of malignant cells [12].

In T-cell lymphoma, the addition of etoposide to the CHOP backbone has had contradictory results. Schmitz *et al*. [13] demonstrated that this approach improves survival for ALK-positive ALCL patients, but not for other subtypes of PTCL. However, a recent study was not able to show similar results [14]. As many patients in our study were treated with CHOP, we suppose that the inclusion of etoposide has contributed to the overcome of worse prognosis associated with TOP2A overexpression, with exception to the PTCL-NOS group.

For PTCL-NOS, univariate analysis revealed that overexpression of *CCNA2, IKBKB,* and *CHEK1* conferred shorter 3-year OS. These results were not surprising, as *IKBKB* encodes a protein that phosphorylates the inhibitor of the inhibitor/NF-kB complex. Consequently, NF-kB is activated, triggering strong proliferative and inflammatory responses in the cell [15]. The constitutive activation of NF-kB in various types of solid tumors and hematological cancers, such as Hodgkin lymphoma (HL) and multiple myeloma, has been previously described [16, 17]. NF-kB-related genes cooperate to increase tumor progression, cell migration, inflammation, and vascular proliferation, and have been used as targets in cancer therapy [18, 19]. In contrast to our data, one study that analyzed the expression of 109 genes associated with NF-kB pathways in 62 PTCL patients identified two different subgroups of PTCL, and the subgroup presenting up-regulated NF-kB genes was associated with better OS [20].

Interestingly, as CHEK1 is known as a DNA-damage response (DDR) coordinator [21], individuals carrying inactivated mutations in this gene are more susceptible to developing many types of cancer, as seen in Li Fraumeni syndrome patients. In our study, the expression of CHEK1 was associated with worse prognosis for PTCL-NOS and better survival for ALK-negative ALCL patients. Physiologically, CHECK1 activation triggers the cell cycle checkpoint, cell cycle arrest, and DNA-repair mechanisms, as well as cell death to avoid formation of damaged cells [22].

Recently, CHEK1 was tested as a target for anticancer drugs. Ravi *et al*. showed that the small molecule proteasome inhibitor ixazomib inhibits tumor growth in xenograft T-cell lymphoma, and HL in mice by downregulating *CHEK1* and *Myc* [23]. The authors’ conclusion was that *CHEK1,* as well as *Myc,* work as targets for ixazomib in cancer, and chemotherapy protocols including anti-CHEK1 drugs may represent a new approach for T-cell lymphoma therapy. They also showed that inhibition of CHEK1 activates caspase-dependent apoptosis in mouse models of B-cell lymphoma [23].

Hoglund *et al*. described the ability of Myc to induce *CHEK1* transcription via a mechanism independent of the DNA damage response, such as ATM and p53 [24]. Furthermore, as we found that *CHEK1* was related to a shorter OS for nodal PTCL in the multivariate analysis, and for PTCL-NOS in the univariate analysis, we hypothesize that ixazomib should be tested in these diseases**,** as proposed by Boonstra *et al*. in his article [25]. As the treatment options available for T–cell lymphoma are scarce, we consider that clinical trials with new drugs are urgently needed for these highly aggressive groups of diseases with very poor prognosis.

The contradictory results for CCNA2 and CHECK1 in the whole cohort/PTCL-NOS and ALK-negative ALCL subgroups are in agreement with theories that gene effects are dependent of the interaction of intracellular networks of molecules, cell connections, the microenvironment, and epigenetic factors [26]. Furthermore, *CHEK1* is related to different prognosis in distinct types of cancer, and even in particular subgroups of the same tumors [27].

The reproducibility of gene expression profiling studies is strongly dependent on the amount of hypotheses and variables tested. Considering a large number of variables being analyzed, and a small population size, higher are the possibilities of finding false positive correlations and false positive results [28]. In addition, few studies have focused on demonstrating the role of genetic and epigenetic issues in PTCL; most of them have shown broad heterogeneity of results with low reproducibility.

PTCL is a rare disease of difficult diagnosis and hard to determine different histological subgroups. Consequently, the detection of recurrent genetic abnormalities is challenging. Furthermore, tumor sample quality must be high for PTCL diagnosis, since a broad IHC panel is often needed to establish an accurate diagnosis [5] Currently, there is not a gold standard microarray platform for gene expression analysis that is universally recommended in the literature. Researchers worldwide have been applying different sets of methods for this goal.

Surprisingly, ALK-negative ALCL patients presented higher survival rate than ALK-positive ALCL patients. At the time of study enrollment, our protocol did not incorporate consolidation with ASCT for high-risk ALK-positive ALCL in first CR, but only for ALK-negative patients in first CR/PR independent of the risk index. As half of our population of ALK-positive ALCL patients had a high risk IPI, we infer that this different approach for treatment could partially explain the contradictory outcome. Recently, d’Amore *et al*. showed that, inside the nodal PTCL, the ALK-negative ALCL subgroup benefits greatly from ASCT, with a 5-year OS of 70% versus 47% and 52% for PTCL-NOS and AITL, respectively [29].

Although ALK protein has been associated with a good prognosis, it is not always seen as an independent prognostic factor [30, 31]. Other authors have demonstrated that the prognosis related to ALK is dependent on other variables, such as age, IPI, CD56 expression, and B2 microglobulin [32–34]. Recently, Sibon *et al*. [34] demonstrated in 138 cases of ALCL that patients younger than 40 years of age presented similar OS regardless of ALK status. Thus, the high proportion of young patients in our ALK-negative ALCL subgroup may also explain the better outcomes observed in this group.

De Leval *et al*. and Piccaluga *et al*. demonstrated that many genes involved in angiogenesis are overexpressed in AITL, especially VEGF1 [35, 36]. In AITL, high levels of VEGF1 are associated with bone marrow infiltration by tumor cells, cutaneous involvement, and high risk IPI. These authors suggested that VEGF1 could be used as a biomarker of PD in AITL [37]. We did not obtain similar results, as VEGF1 expression was not associated with prognosis in our AITL population. However, in univariate analysis, we found that overexpression of *VEGF1* was associated with worse PFS in ALK-negative ALCL. Moreover, we question whether *VEGF1* should be explored as a molecular target for therapy in ALK-negative ALCL.

In agreement with the literature, the most common subtype of PTCL in our study was PTCL-NOS. However, we also found a higher prevalence of ALCL than AITL, which is the second most prevalent PTCL in Europe [5]. Interestingly, the higher prevalence of ALCL was in accordance with North American epidemiology, where ALK-positive ALCL predominates in comparison to AITL [38]. However, the lower prevalence of AITL in our population could be partially explained by the difficulty of diagnosis. AITL presents peculiar morphological features that require adequate specimens, preferably obtained by excision biopsy to allow identification of the arboriform aspect of the vases, of the uneven arrangement of FDCs, and of the paracortical expansion of EBV-positive large B immunoblasts [3].

We observed a high rate of discrepancy between previous diagnosis and after the centralized review in 10.3% (9/87) of cases. This was similar to what was previously described by the International T-cell Lymphoma Project, which reported a 10.4% discrepancy [5]. These data suggest that more specific diagnostic markers are necessary to improve the accuracy and reproducibility of the subtypes of PTCL classification. Moreover, in the histology review, we added more IHC markers to identify follicular helper T-cells, the neoplastic cells of AITL, as well as CXCL-13, PD-1, and ICOS. This approach allowed us to identify more cases of AITL, and more stringent morphological criteria allowed us to identify hallmark cells with strong expression of Ki-1 (CD30), which was essential for differentiating ALK-negative ALCL from CD30+ PTCL-NOS.

In conclusion, we found that overexpression of *CCNA2* and CHECK1 protein are associated with worse OS in nodal PTCL. However, high expression of *CCNA2* and *CHEK1* had an opposite effect on the prognosis of PTCL-NOS and ALK-negative ALCL. No gene or protein studied was able to discriminate subgroups of nodal PTCL. Future studies with more patients are necessary to confirm these findings and improve our knowledge of PTCL.

## MATERIALS AND METHODS

### Study population

After local ethics committee approval, 94 patients between 16 and 86 years old with a diagnosis of predominantly nodal PTCL, treated at a reference center for cancer in Brazil, were retrospectively evaluated from January 2000 to December 2015. Patients with primary cutaneous CD30+ T-cell lymphoproliferative disease, heart failure grade III/IV, unavailable paraffin blocks or medical records were excluded.

Baseline clinical and disease features, including age, gender, Ann Arbor stage, number of extranodal sites involved with lymphoma, LDH dosage, Eastern Cooperative Oncology Group (ECOG)performance status, B symptoms, and bulky disease (tumor size ≥ 10 cm or over one-third of the cardiothoracic index) were taken from medical records by a specific researcher. HBV, HCV, HIV, and HTLV-1 serology, a comprehensive biochemical panel, including kidney and liver function, echocardiogram, bone marrow biopsy, and neck, chest, abdomen, and pelvis computed tomography (CT) were performed at diagnosis. The patients were stratified in clinical stages I, II, III, and IV according to Ann Arbor criteria [39].

The interim response, after the fourth cycle of chemotherapy and the final response at the end of treatment, was evaluated using the Cheson 2014 criteria [40]. The IPI and PIT scores were calculated for the cohort as described previously [9, 10]. ALK-positive ALCL patients were treated with six to eight cycles of CHOP-21 (cyclophosphamide 750 mg/sqm IV on day 1, vincristine 1.4 mg/sqm [max 2 mg] IVon day 1, doxorubicin 50 mg/sqm IV on day 1 and prednisone 100 mg/day PO from day 1 to 5). The majority of ALK-negative ALCL, PTCL-NOS, and AITL patients were treated with six to eight cycles of CHOEP-21 (CHOP plus etoposide 100 mg/sqm IV days 1 to 3).

Patients with bulky disease, sinus, bone, testis, breast, and Waldeyer’s ring involvement underwent field radiotherapy with 3600 cGy at the end of chemotherapy. Autologous hematopoietic stem cell transplantation (ASCT) was indicated for consolidation in first line therapy for patients in complete remission (CR) or partial remission (PR), except for ALK-positive ALCL and patients >65 years of age or with clinical contraindication. ASCT was indicated for ALK-positive ALCL only in second CR or PR. After the end of treatment, patients were monitored by clinical and laboratory exams every 2 months in the first year, every 3 months in the second year, every 4 months in the third year, every 6 months in the fourth year, and annually after the fifth year.

### Histology and immunohistochemistry

Two hematopathology experts reviewed the tumor histology according to the WHO criteria [3]. Four-millimeter sections from formalin-fixed paraffin-embedded (FFPE) tumor samples obtained at diagnosis were stained with hematoxylin-eosin (HE), followed by an initial immunohistochemistry (IHC) panel with monoclonal antibodies (MoAbs) CD20 for B-cells, CD3 for T-cells, Ki-1 (CD30), and cell proliferation marker Ki-67 (Table 4).

**Table 4:** Characterization of monoclonal antibodies used in IHC

Monoclonal antibody	Brand	Clone	Dilution
CD2	Monossan	AB75	1/200
CD3	Dako	F7.2.38	1/500
CD4	Spring	SP35	1/400
CD5	Cell Marque	SP19	1/300
CD7	Novocastra	CD7-272	1/3000
CD8	Dako	C8/144B	1/800
CD10	Novocastra	S6C6	1/2000
CD20	Dako	L26	1/1000
CD30	Cell Marque	Ber-H2	1/1000
CD45	Dako	2B11+PD7/26	1/2000
CD56	Cell Marque	123C3-D5	1/400
Ki67	Dako	K55	1/1600
ALK1	Spring	SP8	1/400
EBV	Dako	C5’1, C5’2, and C5’3	1/3000
CD21	Novocastra	2G9	1/800
CD23	Biocare	1B12	1/1000
CD31	Dako	JC/70A	1/100
CD34	Dako	QBEand-10	1/2000
ICOS	Abcam	SP98	1/100
CXCL13	Abcam	Ab112521	1/300
PD-1	Abcam	NAT105	1/1000

If HE morphology was suggestive of AITL, as described by Swerdlow *et al*. [3], including atypical and polymorphic lymphoid infiltrate with exuberant vascular proliferation and irregular arrangement of follicular dendritic cells (FDCs), the IHC panel was expanded to search for follicular T-helper lineage cells (CD10, BCL-6, ICOS, PD-1, and CXCL13), vascular components (CD31 and CD34), FDCs (CD21, CD23 and CD35), and Epstein-Barr virus (LMP-1 or EBER for *in situ* hybridization) (Table 4).

If HE was suggestive of ALCL, with atypical lymphoid infiltrate of anaplastic large cells or CD30-positive “hallmark cells”>70% [41], IHC for PAX-5, CD15, and ALK was added to exclude Hodgkin lymphoma. PTCL-NOS was diagnosed by exclusion in cases exhibiting atypical and diffuse small to medium lymphoid infiltrate, lacking criteria for characterization of any other category of nodal PTCL [3], and after an extensive IHC panel for T-lymphoid markers with CD2, CD4, CD5, CD7, CD8, TCR α/β, and TCR γ/δ (Table 4). All cases categorized as PTCL-NOS consistently presented negative serology for HTLV-1.

**Figure 5:**
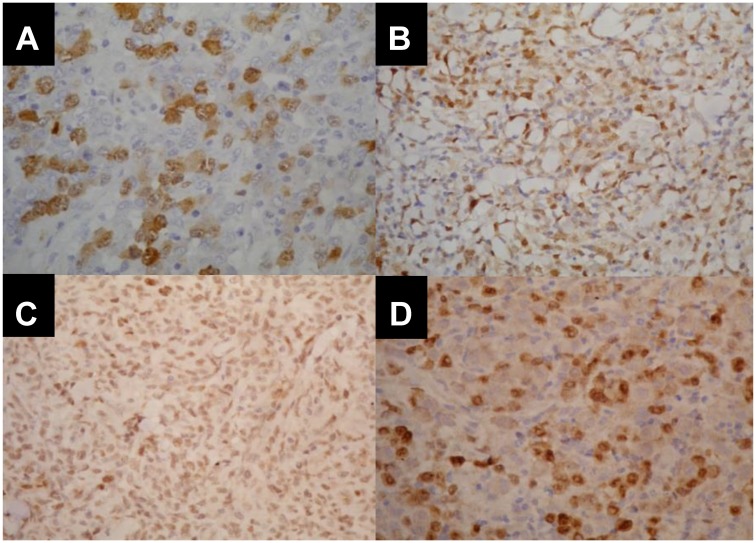
Immunohistopathological pattern of CCNA2, TOP2A, NFkB and CHEK1 expression in different histopathological subtypes of nodal PTCL. (**A**) Cyclin A2 nuclear positivity in 80% of ALCL/ALK+. (**B**) Topoisomerase 2 nuclear positivity in 70% of ALCL/ALK-. (**C**) Nuclear expression of NF-kB in 90% of ALCL/ALK-. (**D**) CHEK1 positivity in 70% of ALCL/ALK- nuclei.

Immunohistochemical staining was performed to evaluate TOP2A, CCNA2, CHEK1, and NF-kB1 protein expression. Manual staining was performed on 4-mm sections obtained from FFPE samples, using MoAb for TOP2A (clone Ki-S1, 1/4000, Dako, USA), CCNA2 (clone 6E6, 1/100, Abcam, USA), CHEK1 (clone DCS-310, 1/200.000, Sigma-Aldrich, Germany), and NF-kB1 (polyclonal, 1/3500, Abcam, USA). The number of positive cells was determined in 20 fields of 200 cells under an optical microscope (40X objective). The positive score was determined as the percentage of positive tumor cells using a semi-quantitative method in 10% increments. Two distinct observers analyzed the glass slides (Figure 5).

### Molecular biology

Total RNA was extracted from FFPE tissues (4-μm-thick) using the Qiagen FFPE RneasyKit (Valencia, CA, USA) with a modified deparaffinization step [42]. Briefly, sections were deparaffinized by two repeated incubations in xylene for 10 minutes, followed by two repeated incubations in 100% ethanol for 5 minutes, and then washed with distilled water for 30 seconds. After deparaffinization, the remaining steps of RNA extraction were followed according to the Qiagen FFPE RNeasy Kit manual. All RNA samples were analyzed spectrophotometrically (NanoDrop 1000 Spectrophotometer V3.7, Thermo Fisher Scientific, Wilmington, DE, USA).

cDNA was synthesized from 1.0 μg of total RNA using the High Capacity cDNA Reverse Transcription Kit (Applied Biosystems, Foster City, CA, USA) according to the manufacturer’s protocol. The integrity of the cDNA template was evaluated using the endogenous *GAPDH* (cat: 4352665) and *GUSB* (cat: 433767) TaqMan Gene Expression Assays (ThermoFisher). Subsequently, qRT-PCR was carried out for *CCNA2* (Hs00996788_m1), *TOP2A* (Hs01032137_m1), *CHEK1* (Hs00967506_m1), *VEGF1* (Hs00173626_m1, *NFkB1* (Hs00765730_m1), and *IKBkB* (Hs00233287_m1).

Standard curves were prepared using total RNA from the KG1 cell line (ATCC-CCL246 Manassas, VA, USA). The 5× 10-fold serial dilutions were prepared from the original cDNA stock and qRT-PCR assays were performed on a 7500 FAST Real Time PCR System (Applied Biosystems, Foster City, CA, USA) using Applied Biosystems Taqman^®^ Gene Expression Assays (Applied Biosystems) according to the manufacturer’s instructions and TaqMan^®^ Universal PCR Master Mix (Applied Biosystems). Expression ratios were calculated based on relative quantification. Samples with *GAPDH* or *GUSB* Ct values > 34 were not included. The sample acceptance criteria was based on the Minimum Information for Publication of Quantitative Real-Time PCR Experiment (MIQE) guidelines [43] and the determined PCR efficiency for each assay using FFPE for GAPDH, GUSB, and all tested genes (all 96.5%–99.0%)

### Statistical analysis

The univariate analysis to assess the association among categorical variables was performed using the Mantel-Haenszel chi-square test (mhodds). A Cox univariate analysis was performed to estimate the association between categorical variables and survival curves. The log-rank test was used to compare survival curves and to verify the association between categorical variables and survival curves. Variables that were significant in the univariate analysis were tested in a multivariate analysis using proportional hazard model stepwise backward selection to establish independent variables.

OS and progression-free survival (PFS) curves were estimated by the Kaplan-Meier method. OS was measured from the date of diagnosis to the date of death from any cause and was censored at the date of the last follow-up. PFS was assessed from the date of the diagnosis to the date of progression, death from any cause, or the last follow-up. The cutoff points for protein expression were determined by the association between their median expression, and sensitivity and specificity were estimated using receiver operator characteristic (ROC) curves. Statistical analyses were performed using STATA 12.0 software. *P*-values ≤ 0.05 were considered statistically significant.

## 

### Ethics approval

This study was performed in accordance with the Declaration of Helsinki and approved by the Faculdade de Medicina da Universidade de São Paulo (FMUSP) Ethics Committee.
